# Job crafting motives and strategies to increase work-related well-being among healthcare employees

**DOI:** 10.1177/10519815251353454

**Published:** 2025-07-10

**Authors:** Ellen Jaldestad, Andrea Eriksson, Göran Jutengren, Åsa Tjulin

**Affiliations:** 1 Division of Ergonomics, Department of Biomedical Engineering and Health Systems, KTH Royal Institute of Technology, Stockholm, Sweden; 2 Department of Welfare, Management and Organization, Østfold University College, Østfold, Norway; 3 Department of Health Sciences, Mid Sweden University, Östersund, Sweden

**Keywords:** healthcare, interview, patient care team, public sector, work-life balance, work engagement

## Abstract

**Background:**

Employees often change and adapt work to increase the fit of their own goals and needs, and resources and demands in work. Crafting a job in this manner can promote well-being at work.

**Objective:**

This study aims to explore different job crafting strategies that healthcare employees engage in to increase their perceived well-being at work and the motives behind these strategies.

**Methods:**

Semi-structured interviews were conducted with 16 healthcare employees, including one dental nurse, assistant and registered nurses, and occupational therapists. Interview data was analysed thematically.

**Results:**

The informants in this study all engaged in crafting strategies that were derived from more than one of four emerging motives. These motives were to craft for their development, for a common good, for meaningfulness in work, and to craft for manageability. Crafting strategies within the different motives included adding tasks beyond the clinical work, developing relations for collaboration with colleagues from other healthcare professions, involving patients when planning their daily work, and developing templates to optimize work. One other strategy to make work more manageable was to choose, at times, to craft less or to not craft at all.

**Conclusions:**

Job crafters engaged in different crafting strategies, derived from different motives, which seem to change depending on their current work-, and personal situation. Even though an inner drive for development seemed to overcome constraining working contexts, it is suggested that health-promoting job crafting should be organized through the promotion of ideas and employee-driven initiatives, as well as through cross-professional collaboration.

## Introduction

Employees rarely perform their jobs strictly according to the formal ‘protocol’. Rather, they commonly adjust the work procedures to increase the fit of their resources and preferences. This kind of work performance adjustment has been recognized as job crafting. Job crafting was originally defined as “*the physical and cognitive changes individuals make in the task or relational boundaries of their work”*.^1, p. 179^ According to this definition, individual employees craft their jobs, resulting in altered work meaning and work identity. Initially, three types of job crafting were described: (i) *task crafting* includes adding new or adjusting current job tasks – for example, asking for responsibilities outside the traditional job description; (ii) *relational crafting* can be exemplified by getting to know your colleagues for social support or knowledge exchange; (iii) *cognitive crafting* includes actively changing the way you think about work – for example, to consider the tasks conducted, even boring ones, as important parts of a bigger whole.^1^ Further, the job crafting concept was framed within the job demand-resources model (JD-R), and three ways of crafting demands and resources in work, to increase person–job fit, were identified.^2^ Those were (i) *increasing job resources* (social and structural); (ii) *increasing challenging job demands*; and (iii) *decreasing hindering job demands*. The aim of job crafting is thus to align individual preconditions, needs, and abilities on the one hand, and job-related demands and resources on the other.^1–3^

Within the job crafting theory, motivation plays a crucial role in individuals engaging in job crafting activities and strategies.^1^ A perceived job–fit imbalance in work can, for example, trigger employees to engage in job crafting activities.^2,4^ There must also be perceived ‘room for crafting’ within the current work context. Previous research indicates that personal characteristics interplay with organizational conditions to either facilitate or hinder job crafting. On the individual level, being motivated and engaged in work and having an inner drive for development have been connected to job crafting in general^5,6^ as well as within the healthcare sector.^7–11^ These characteristics have been connected to *approach*- and *promotion-oriented* crafting strategies, such as adding new and challenging tasks and crafting for increasing resources in work. In contrast, some negative characteristics, such as workaholism and burnout, have been connected to *prevention*-*oriented* crafting strategies aimed at reducing hindering demands (e.g., reducing the number of tasks) and increasing structural resources (e.g., asking for more time to conduct tasks).^12^ Work experience (i.e., years in the job) and level of skills have also been connected to job crafting among healthcare employees; with more work experience come more job crafting activities.^13,14^ On the workplace level, autonomy and task independence, as well as people-oriented leadership approaches that seek to empower, and build supporting relations with employees, have been found to promote job crafting among employees.^2,14–17^ So too have social capital within the workgroup (e.g., mutual trust, and openness to new ideas).^18^

In this study, we intended to focus specifically on job crafting strategies that were perceived to promote healthcare employees’ health and well-being at work, as well as motives to craft their jobs accordingly. That is, we explored job crafting strategies that were said to contribute to, for example, motivation and work engagement, or manageability and meaningfulness, rather than reactive strategies resulting in negative outcomes, such as stress, overload, and neglecting certain tasks.^19,20^ Galvin and Todres^21^ presented an approach to health and well-being as something “*much deeper and more complex than just the absence of illness*”, and that individuals can experience health and well-being in different ways. In this study, we assume that what makes one person feel good at work is not necessarily the same for a colleague. Galvin and Todres^21^ define in this context different experiences of subjective health within two dimensions: *mobility* and *dwelling.* According to their framework, people who perceive health in relation to movement (i.e., *mobility*) are people who enjoy ‘being on the move forward’ and who get energy from ‘adventures’ and widening their horizons. This could be connected to people who experience well-being in work when they can challenge themselves to learn, grow, and develop within their work, for example, by engaging in approach- and promotion-oriented job crafting strategies. For others, according to Galvin and Todres,^21^ health and well-being in work are instead connected to a sense of ‘at-homeness’, rootedness, and settling (i.e., *dwelling*). For these people, work-related well-being could be linked to organizational stability and to being able to manage a current work situation without unexpected events. In relation to job crafting, preventive strategies, such as reducing the number of tasks to keep work manageable, and cognitive crafting strategies, such as focusing on the meaning of the current work, could be linked to dwelling for health and well-being. Galvin and Todres^21^ also unite the two dimensions in a *dwelling-mobility* dimension, since people can perceive sources for well-being differently over time and in different situations.

It should be acknowledged that this study was conducted within public healthcare, which is a working context with different challenges in terms of being able to work with maintained health and well-being, such as stress, heavy workload, and staff shortages.^22^ Thus, healthcare employees’ perceived well-being in work, as well as their strategies to promote their well-being, interplay and may change due to the challenges they face within their working context. Job crafting strategies from a health-promoting perspective have earlier been studied in private sectors.^20^ Also, job crafting has been extensively studied within the healthcare sector in the last couple of years, often focusing on the connection between job crafting and work engagement among different professional groups.^23–26^ Being engaged in work has also been connected to healthcare employees’ perceived well-being in work.^8,27^ So too have job satisfaction and perceived meaningfulness among employees, which both have been found to be outcomes of job crafting.^1,3,4^ Thus, engaging in job crafting activities can increase employees’ well-being. Interventions aiming for job crafting in different occupations have been shown to have impact on job crafting behavior as well as self-efficacy.^28^ Since employees who are engaged and feel well contribute more to the production and overall performance, and tend to stay within their current position, job crafting has positive effects on the organization.^2,27^ Although job crafting usually has positive effects, some negative consequences have been identified. With more crafting, more stress and overload can follow.^29^ In addition, a person might craft their way too far out of the work-related boundaries, for example neglecting troublesome tasks or clients.^19^

To the best of our knowledge, there remains a lack of qualitative studies focusing job crafting strategies that promote well-being, and the motives behind them, among public healthcare employees. Drawing from this background, our aim in this study was to explore different job crafting strategies that healthcare employees engage in to promote well-being at work, and the motives behind these strategies.

## Methods

### Study design

An abductive qualitative explorative research designs was used to gain in-depth insight to different job crafting motives and strategies that promote work-related well-being among healthcare employees: semi-structured interviews were conducted with employees from different professions and with different lengths of work experience, and analysed using abductive thematic analysis.^30–33^ The COREQ checklist (Consolidated Criteria for Reporting Qualitative Research)^34^ was considered in the study design. The data collection was conducted as part of a larger research project within Swedish public healthcare and included interviews with both employees and managers. In this study, an employee perspective was chosen, thus only the interviews conducted with healthcare employees have been analysed. The interviews with the informants in this paper have also been used in another study carried out within the research project, with the aim of identifying antecedents of health-promoting job crafting among healthcare employees. The studies had different aims and were analysed separately.

### Sampling and data collection

The informants in this study included 16 healthcare employees who represented the following healthcare professionals: one dental nurse; four assistant nurses; five registered nurses; and six occupational therapists. Four informants were men and 12 were women, and the work experience ranged from six months to 43 years in their current profession. The informants were recruited from five different care workplaces within Swedish public healthcare, located in three separate healthcare regions. The workplaces included one dental care clinic, one surgery department, one surgery care department, one radiology department, and one occupational therapy department. The participating workplaces were recruited from professional networks in the research group, and all of them were known to us as being active in developing their work environment – for example from participating in previous research projects (e.g., health-promoting leadership). This selection of departments was chosen in line with the study aim, with the intention to include workplaces where employees had experiences from health promoting activities. To recruit informants, clinic managers and ward managers were initially contacted via e-mail with an invitation to participate in the study. If accepted, employees from different professional groups, and with different lengths of work experience, were requested to participate. This was to provide empirical data from a range of professions and experience as broad as possible. Employees were invited to participate by their closest manager. Their participation was voluntary, albeit known to the immediate superior.

The interviews were conducted at the employees’ workplaces, in separate rooms with closed doors, and the informants were allowed to take the time needed without being disturbed. All interviews were recorded and lasted about one hour. A master's student who wrote her thesis within the current research project interviewed the occupational therapists while the first author of this article (a female PhD student) conducted the interviews with the remaining informants. The interviews were conducted between 2017 and 2019. Semi-structured interviews allowed informants to speak freely about their experiences and perceptions of the research area, and the interviewer could explore certain areas of interest in more depth.^35^ The concept of ‘information power’^36^ guided the sample size in this explorative study. Rather than focusing the number of informants, the sample was selected to provide sufficient information value to support the study's aim. Additional individual interviews were conducted until an adequate breadth and depth of information was achieved in relation to the study's purpose, consistent with Malterud's concept of “information power”. Following Low's discussion,^37^ the analysis aimed to reach “conceptual rigor”^34^^, p. 136^ rather than traditional saturation, which focuses on the absence of new information, themes, or categories. Instead of saturation in this sense, the analysis was anchored in a breadth of previous studies and job crafting literature, focusing on the different motives and strategies described by the informants. Interview protocols with topic areas derived from previous research on job crafting and health-promoting work were developed to guide the interviews, and the informants were for example asked about their *different strategies to change and adapt work*; *what they thought motivated and facilitated those strategies*; and *perceived outcomes of the strategies*.

### Data analysis

All recorded interviews were transcribed verbatim and thereafter imported into NVivo. To answer the aim of this study, a reflexive thematic analysis was chosen.^30,31^ Questions that guided the analysis process were: *what motives do the informants describe to craft their jobs?; how do the informants craft their jobs?; in what way are the crafting strategies perceived as promoting their work-related well-being?.*

The analysis process was iterative and comparative in nature. To become familiar with the content, the first author of this article read and listened to the interviews repeatedly. Interview data were first interpreted openly and inductively when generating the first draft of concrete codes from each transcript. Secondly, the codes were deductively interpreted from our pre-understanding of theories on job crafting and work-related well-being.^30,31^ Initial themes included both concrete and abstract subthemes, as well as both motives for job crafting and job crafting strategies. From this phase, each informant's codes could be found in more than one theme. That is, one informant explained how different motives led to different crafting strategies. Mind maps and tables were used to sort and adjust themes and subthemes. Thirdly, the initial themes were revised in an iterative process including several meetings and discussions with all authors of this article. Themes and sub-themes were regularly tested on the empirical data (the interview transcripts), and adjusted when needed. For example, two themes were merged into one, and sub-themes that previously occurred in more than one theme were analysed further to find their most suitable theme. In the analysing process, four different themes of motives to craft for work-related well-being were generated: to craft for their development, common good, meaningfulness in work, and for manageability. These motives led to different crafting strategies among the informants, which are seen in the subthemes. See Table 1 for a summary of the analysis process.

**Table 1.** Flowchart of the analytical process.

Examples from the transcripts	Examples of subthemes / Crafting strategies	Themes / Motives to craft
I have asked to be in [a larger hospital] … to be trained. Because after one year now … I need to get a little more experience to be able to work in this place because there are so few of us and then you need to have a little more [experience].	Asking for new assignments/developing opportunities Planning exchange with larger/other hospitals with similar fields	To craft for their development
If someone calls and asks for help, then I do not want to say no but: ‘It's good that you ask for help, I can help you’. Then maybe this person can learn how to do this on their own next time.	Making themselves available for other departments Transfer/exchange knowledge	To craft for a common good
I’d had it in my mind that I would make a home visit. But when I got there, he was waiting for me outside his door. And then he said … “It's me you're going to meet, but I'm not going to let you in” … Then I was completely taken aback… After all, we are always in a home or garden or something like that. But he didn't want to … But he had some errands to run, so we were in town for a whole morning. … He showed that he would like to participate, but on his terms. And in retrospect, I can think: “Wow! That's good!” … healthcare must be for the patients … he should be allowed to decide. I am not going to stand there and insist that we have to go up to his apartment.	Involving patients in assessments and treatments – adapting assessments to better suit the patients and their needs and conditions Planning the day to better fit the patient's needs and desires	To craft for meaningfulness in work
I usually take a piece of white paper and draw a long line, like a timeline … and then I know the patient's date of birth. Then I ask them to say the date of birth first, and then we see if we can fill that line up to today.	Creating templates for patient work (e.g., assessments, patient communication)	To craft for manageability

### Ethical consideration

The study was part of a larger project on how learning processes at different organizational levels interplay with employee health which was approved by the Ethical Review Board in Stockholm (Dnr. 2014/1883-31/5). The informants were informed both verbally and in written text about the study and signed their informed consent to participate. They were informed that they could stop the interview at any time. Since managers recruited informants it was considered that managers’ knowledge of who participated in the study may have impacted informants’ willingness to provide, versus withhold certain information, due to fear of being negatively affected by their participation.^38^ To increase confidentiality to the greatest extent, collected data were handled in line with recommendations of good research ethics.^39^

## Results

The emerging motives for job crafting to promote work-related well-being among the informants were (i) to craft for their development; (ii) to craft for a common good; (iii) to craft for meaningfulness in work; and (iv) to craft for manageability. Two of the themes (i and iv) had an individual perspective, meaning that the job crafter engaged in strategies that mainly favored themselves. In the second theme (ii) the job crafters also had an organizational perspective: the crafting strategies within this theme aimed to improve work both for themselves as well as others. The third theme (iii) included crafting strategies that favored both the informants and their patients.

All informants engaged in more than one crafting strategy; one specific motive to craft their job led to one kind of crafting strategy, whereas another motive to craft their job led to other crafting strategies. See Table 2 for a schematic view of which themes were expressed in each informant's job crafting. There were also informants who – for various reasons – at times did not craft at all, or crafted considerably less than before. These strategies, and their connections to work-related well-being, are included in theme four (iv) to craft for manageability below.

**Table 2.** Summary of job crafting expressions within the analytical themes.

INFORMANT THEME	DN	AN	AN	AN	AN	RN	RN	RN	RN	RN	OT	OT	OT	OT	OT	OT
i)		x	x		x	x	x	x	x	x	x	x		x	x	x	x
ii)		x			x	x					x	x	x	x	x	x	x
iii)		x		x		x	x	x	x	x		x	x	x	x		x
iv)		x	x	x	x	x	x	x	x	x		x	x	x	x	x	x

DN: dental nurse; AN: assistant nurse; RN: registered nurse; OT: occupational therapist.

i) to craft for their own development; ii) to craft for a common good; iii) to craft for meaningfulness in work; and iv) to craft for manageability.

### To craft for their own development

The first job crafting motive holds an individual perspective in that crafting strategies were connected to the informants’ own well-being in terms of engagement, motivation, and job satisfaction through learning, and personal and professional development. Some informants expressed that they had support from their colleagues and managers when engaging in crafting strategies for their development. Others, however, explained that they lacked support from peers and managers when they wanted to develop further in work. Some informants had changed their professional paths completely after a longer career in different fields and explained that they left their previous security to fulfill new dreams. Some informants expressed that they constantly looked for things to develop alongside their clinical work – for example, areas they wanted to learn more about:

*Every time you feel comfortable [in work], you are probably looking for your level of incompetence … What else can I do? Is there anything I can develop within?*

Among informants with little experience in their current work, the most common crafting strategy within this theme was to ask for new or extra assignments. Based on their interest, informants could also choose to expand their responsibilities beyond clinical work, such as responsibility for managing emergency trolleys and purchasing supplies.

Some of the more experienced informants explained how they planned to or already had strategies for further training in their field. For example, one occupational therapist chose to work close to a physician, and sometimes asked to be an observer during the surgery of their future patients, to learn more and know more about their patients. The same person also planned for professional exchange with another hospital with similar activities as in their department. One registered nurse already had arranged an exchange with a larger hospital with more advanced surgery. Both individuals expressed a desire to learn more and gain more experience within their field:

*I have asked to be in [a larger hospital] … to be trained. Because after a year now … I need to get a little more experience to be able to work in this place because there are so few of us and then you need to have a little more [experience]*.

There were also informants who had had the opportunity to shape their specialized roles. They had grown trustful relationships with their managers, and when their desire to develop further within their field became strong enough to form their roles, they found the room to do so without changing employers or careers. Developing relations with their managers was considered a strategy to enable further development within their current profession among several informants:

*So, I designed it [the specialized role], agreed with the clinic manager, and went to my [ward] manager and said I want to work like this. Showed the need for it. Then I got it*.

### To craft for a common good

In comparison to the job crafting motive and strategies presented above, this theme holds an organizational perspective with examples of how the informants engaged in crafting strategies to improve work for both themselves and their colleagues. Examples of strategies to improve social relations in the workplace were to plan and promote activities such as celebrating birthdays and certain occasions, and socializing activities in their spare time. Crafting for the common good also included organizing and optimizing work for more than themselves; for example, to improve cooperation and the quality of care within the departments.

Some of the participants actively sought support from their colleagues, both within their profession and among others. Discussing and collaborating around a common patient were said to contribute to cross-professional collaboration, even when there was no formal organization for that kind of teamwork.

For some informants who worked in more than one department, it was important to make themselves available and to become a natural part of the different teams. These individuals also expressed that they wanted to transfer their specialist knowledge to others – for example, to optimize work for themselves and their colleagues by teaching them how to conduct certain procedures on their own:

*If someone calls and asks for help, then I do not want to say no but: ‘It's good that you ask for help, I can help you’. Then maybe this person can learn how to do this on their own next time*.

The participating occupational therapists all had different ‘home departments’, meaning that they were the only occupational therapists in that specific department. To them, it was particularly important to build professional relationships, both with other occupational therapists in the hospital as well as other professional groups (e.g., physicians and physiotherapists). They contacted their occupational therapist-colleagues for support, and to exchange knowledge, and arranged to meet their patients together with other professionals:

*Sometimes I join the patient on the visit to the physician so we will have a common understanding, especially those … where I have difficulty progressing, but the physician knows. We can make a plan and see how we should proceed*.

### To craft for meaningfulness in work

Informants engaged in job crafting strategies aiming to make work more meaningful for themselves. One individual said: “*When you leave, you feel a little warm in your stomach; you feel that you have done something important”*. One cognitive strategy to increase perceived meaningfulness in work was to consider one's work as an important part of the care chain, and to know one has done something good for someone else; another was feeling proud of one's work. Informants in all professional categories expressed that they engaged in these kinds of cognitive strategies. They also explained that patients sometimes gave them the feedback they lacked from colleagues and management and that they wanted to make the most of this feedback; doing something good for others, and getting feedback on it, made work more meaningful:

*It [work] can be very tough and sometimes you are very tired because it takes a lot of energy. But it's fun because I think you get the feedback quite quickly … You feel that you are helping someone, even if you sometimes have to do things that are not fun. In any case, we have done a good job*.

However, the crafting strategies in this theme also had a patient perspective, as there were many examples of strategies that aimed to do something good, and increase meaning, for patients as well. A recurrent strategy to achieve this was to involve the patients in the daily work planning: informants planned the day to fit the patients’ requests, needs, and desires – for example by taking into account the patient's energy level, anxieties, and planned visits by relatives. Another example was to involve patients in assessments and treatments; for example, putting the patient in charge of their rehabilitation, and adapting assessments to better suit the patients and their needs and conditions:

*I’d had it in my mind that I would make a home visit. But when I got there, he was waiting for me outside his door. And then he said … “It's me you're going to meet, but I’m not going to let you in” … Then I was completely taken aback…* *After all, we are always in a home or garden or something like that. But he didn’t want to … But he had some errands to run, so we were in town for a whole morning. … He showed that he would like to participate, but on his terms. And in retrospect, I can think: “Wow! That's good!” … healthcare must be for the patients … he should be allowed to decide. I am not going to stand there and insist that we have to go up to his apartment*.

The informants thus focused on their patients both when preparing and conducting work: one informant explained how some ‘detective work’ was done when searching different sources to look for relevant information in favor of the patient. It was prioritized to help the patients more than the informants were obliged to; for example, how to navigate the social security office. The informants also had strategies to communicate with the patients to meet them with respect and to make them feel safe and relaxed, and one way to do so was the way they were talking and (if appropriate) joking with the patients:

*I believe I can read a person quite well. How to talk to a person, in what tone of voice. Therefore, I can probably oscillate between being very kind, humble, and soft, to being quite harsh: “If you don’t do this, then…” [I can be] quite brutal. However, if you have read the person, you can make jokes … sometimes I even swear to make them realize it is serious*.

### To craft for manageability

Among the informants, some expressed motives to craft their work more manageable. Coming from their perspective, they engaged in different strategies to optimize resources at work. Informants chose between active crafting strategies to make work more manageable and to not craft at all when that was perceived to better promote their well-being.

Among the crafting strategies for manageability was to create templates for the work with patients, which made work easier for the informants and released energy for other things. Examples of this were to design templates for the daily planning and standard examinations, and a system for the assistive device inventory. One occupational therapist described how they created a template – a timeline – for their assessments of patients with dementia:

*I usually take a piece of white paper and draw a long line, like a timeline … and then I know the patient's date of birth. Then I ask them to say the date of birth first, and then we see if we can fill that line up to today*.

The most common strategy for increasing manageability was to ask for help and/or support to handle difficult situations, such as challenging patient visits. Building trustful relations with colleagues in their own and other departments resulted in practical help to manage work, in finding confirmation of their work, and in getting understanding from others who worked with similar things. Informants in all professional categories expressed that they engaged in this strategy. Some individuals actively chose whom to ask in different situations – for example in terms of work experience – as well as whom they spent time with during the days to gain energy and to recover:

*You really need these older colleagues. I think that is very important. They were a fantastic security when I arrived. At the same time, it is very nice to have someone who is the same age and in the same phase of life, with whom you can talk about everyday problems*.

Other strategies to gain energy and recover during workdays was to actively balance perceived demands and personal resources, for example by reflecting on the work, ‘choosing their conflicts’ at work, taking breaks, and saying no to others’ requests.

Job crafting was also in the interviews manifested as dynamic in nature; one person could engage in job crafting during one period of time, whereas fewer crafting activities were going on during other periods. Some informants had crafted more before but were now content with work the way it was. They wanted to maintain their stability in work or refine the assignments they had had. For example, some described that they were happy with the development opportunities they had gotten, but for now, they wanted to focus on their current tasks. Another example was how private family matters and work–life balance were motives for fewer crafting activities. Some informants considered themselves to be more ‘laid-back’ at work to save their energy to manage their family situations. Some expressed that they chose to prioritize clinical work during intense periods (e.g., because of uneven patient flow) instead of engaging in job crafting strategies.

*Now I have decided that I'm going to stay here for a while and develop what I already have, so that I don’t take on another thing and then it gets kind of half-good … because it has become a little difficult to find time for everything, to be able to finalize things*.

## Discussion

The aim of this study was to explore different job crafting strategies that healthcare employees engage in to promote well-being in work, and the motives behind these strategies. The informants’ crafting strategies differed in different situations, thus they expressed multiple motives for crafting as well as different crafting strategies to promote their own perceived well-being at work.

When crafting for their own development, the informants engaged in crafting strategies that were said to positively satisfy their inner drive for development, and thus connected to proactive characteristics in their personality – a recurrent antecedent of job crafting.^5^ The job crafting strategies included adding stimulating tasks in addition to their clinical work. The informants who expressed a drive for development and stimulating assignment also seemed to be individuals who experienced health through the dimension mobility, i.e., people who feel well when they have opportunities to widening their horizons.^21^ There were also individuals who not only changed the boundaries of work, as described by Wrzesniewski and Dutton,^1^ but who also crafted their way beyond formal professional roles, for example by forming their own specialized roles. Behind these strategies were motives connected to their inner drive for development, as well as a perceived need for more specialized competence within their home department and/or hospital. This kind of boundary-breaking job crafting was not, however, conducted solely on their own. Even though initiated by the employee, managers were involved in a negotiating crafting process,^29^ which can be defined as more collaborating forms of work crafting.^40,41^ Collaborative forms of work crafting refer to employees negotiating with their managers (or HR or colleagues) to find work arrangements that better meet their development needs, as well as organizational achievement. The fact that employees involve their managers in the crafting process suggests that neither the quality of care nor the stability of work were jeopardized by employees with these motives to craft.^19,40^ Instead, the informants presented an identified personal need (i.e., a desire to develop further) as well as a need for more specialized competence in their home departments. If managers are open to collaborating and promoting the development of employees’ resources to increase their abilities in work, work can become more sustainable.^40^ Mutual trust from managers and an openness to new ideas were said to enable these strategies among the informants, which is in line with previous research that has found social capital within work groups, and people-oriented leadership as job crafting antecedents.^2,14–18^ This indicates an interaction between identified motives and strategies, in this case between the motives to craft for their development, and to craft for a common good. As presented in Table 2, all informants expressed crafting motives and strategies within more than one of the conceptualized themes. Thus, the informants expressed how different motives led to different crafting strategies, and sometimes these were closely connected. It is noteworthy that when crafting for their development, job crafters sometimes did so regardless of working contexts (i.e., supporting or constraining).

In some departments, work was organized in cross-professional teams. In line with previous research, this provided the employees with a more comprehensive picture of the care of the patients, as well as increasing collaboration between the professional groups.^42^ Even when work was not formally organized in cross-professional teams, the employees strived to work close to others with both the same, and other professions. They talked about making use of each other's competencies as well as finding support in each other. For example, it was mentioned by the occupational therapists, who mostly worked on their own, that they developed relations with others from the same and other professional groups, to optimize work for themselves as well as others (i.e., colleagues and patients). Crafting strategies that focus on social relations and social resources could by extension lead to a stronger social capital within work groups, with mutual trust, support, and an openness to new ideas.^43^ Social capital within work groups is in itself an identified job crafting antecedent.^18^ Thus, there seems to be a two-way interaction between social capital and job crafting strategies that increase well-being; social capital may be an antecedent as well as an outcome of employees’ job crafting strategies to promote well-being in work.

To increase meaning in work is a previously identified motive to engage in job crafting,.^1,14^ One way of adding meaning to work is to actively think about how work adds meaning to life.^1,26^ Among the informants in this study, this was exemplified by considering the importance of one's part in a patient's care chain. Another strategy was to involve the patients in their daily work. Even though caring for patients is a major part of the work in healthcare, the informants, however, aligned their crafting strategies with what was considered to have positive effects for the patients. For example, assessments were adapted to better suit the patient's needs and abilities. Thus, their crafting motives seemed to include adding something extra for the patients. Wrzesniewski and Dutton^1^ concluded that there is much to learn from employees who create meaning in work with limited resources. As mentioned above, the public healthcare sector has limited resources. Thus, there can be valuable knowledge about how to increase meaning in work among the informants in this study. A recent study, conducted during the COVID-19 pandemic, found that crafting strategies among general practitioners (GP) increased their meaning of work as well as strengthened work identity when managing work in this unfamiliar and challenging time. The crafting strategies among the GPs during the pandemics did not only focus on their personal development, but primarily on the common good for both the organization of care and patients. Seeking support among peers was common, as well as crafting their tasks to manage the extraordinary daily work during the pandemic.^44^

The informants in our study also talked about how to make work manageable for themselves. This was achieved by crafting in relation to perceived – or limited – resources and demands in work, as described by Tims and Bakker.^2^ One example was to create templates that would make work (e.g., assessments) easier for themselves. Sometimes, motives to make work more manageable, however, also led to less job crafting; to maintain their current perceived well-being in work, the informants chose to craft or not to craft. These results confirm Galvin and Todres^21^ points on that people can perceive sources for well-being differently over time and in different situations. The results also point out that experiences of well-being related to dwelling, i.e., organizational conditions of stability, may be related to coping with high demands in work. It was mentioned by the informants that private matters sometimes interfered with work, or that they prioritized things in work other than their development during intensive periods. Thus, even though the job crafting strategies that the informants engaged in were said to increase their work-related well-being, they sometimes expressed that they instead needed to reduce their strategies to change and optimize work. This can be compared to the findings by Lazazzara, Tims,^29^ who in a meta-synthesis of qualitative job crafting articles, found that crafting strategies can be moved to other domains than the workplace, for example when the context is constraining. Findings by Mayson and Bardoel,^13^ and Harbridge, Ivanitskaya^14^ indicate that more years in the job (i.e., work experience) increases employees’ job crafting. Due to different factors, job crafting may thus change in scope and nature over time. Our focus in this paper has been on job crafting that is perceived to increase employees’ work-related well-being. In line with the shifting in perceived health and well-being presented by Galvin and Todres,^21^ it is suggested that job crafters alter their engagement in job crafting in relation to different factors that affect them, and their perceived well-being, in work. Sometimes, job crafters feel well when actively engaging in job crafting strategies, at other times it is better to dwell.

### Methodological considerations

Different analysis methods were discussed within the research group, before settling with the reflexive thematic analysis.^30,31^ While there were well-thought-out focus areas in the interviews, these were not intended to guide the analysis process. Therefore, it was considered that a thematic analysis was best suited for examining the collected material. The analyses revealed common job crafting motives and strategies among the informants, which were considered interesting to analyse and interpret further through our theoretical lenses and preconceptions of job crafting. One of the strengths of the reflexive thematic analysis is that it allows a theoretical grid and an abductive approach to the analyses which also means that the analyses cannot be conducted in a theoretical ‘vacuum’.^30,31^ The informants were recruited from departments of which several were known to actively strive for a better working environment through different efforts. This sampling strategy is considered both a strength and a limitation in the study design: This may have limited us to include only informants with greater opportunities for health-promoting job crafting strategies, compared to healthcare professionals from other departments, and by that the informants may not have represented the average healthcare employees. However, with the aim of the study being connected to health-promoting aspects in work, this sampling may, on the other hand, have provided important information about how to increase well-being in constraining working contexts.

Another possible limitation with the study design is that the data were collected during two years, which may have affected the study's dependability. The latest data collected during this period were the interviews with the occupational therapists. All data were, however, collected before the outbreak of the COVID-19 pandemic and consequently analysed according to the circumstances prevailing at the time.

A strength of the study design is that all co-authors of this paper, with their different areas of expertise and experiences, were involved in the process of constructing the interview protocol as well as in the data analysis. As mentioned in section 2.2, interviews were conducted until we reached a perceived breadth of informants and depth in the collected material, to reach rigor in the conceptualisations.^37^ We did not aim for a theoretical or thematic saturation in terms of no emerging of new information, themes, or categories. Instead, our aim of the analysis was to provide insight and conceptual rigor regarding job crafting motives and strategies to increase perceived well-being among healthcare employees.^36,37^ The first author of this paper carried out the preliminary analysis. Subsequently, the group of co-authors discussed the results. Further analyses were conducted by the first and last authors of the paper. The final analysis and conceptualisation of themes was carried out together with all co-authors before the final results were established. Thus, the study design and analysis process are considered to increase the quality and trustworthiness in alignment with quality criteria of qualitative research, i.e., credibility, dependability, confirmability, and transferability.^32,45^

### Practical implications

Some practical implications that draw on the transferability on this study include the following: when striving to promote employee well-being, it is suggested to actively organize the facilitating of job crafting. On the workplace level, close collaboration between employees from both the same and different professions seems to motivate job crafting, for example when working in formal cross-professional groups. An openness to new ideas, as well as promoting employee-driven initiatives may also facilitate crafting strategies that positively affect both employees and the organization.

### Conclusion

When crafting for well-being in work, job crafters engaged in different crafting strategies, derived from different motives. Both motives and strategies seemed to change depending on the job crafter's current situation and working context. To have an inner drive for development, however, seemed to overcome constraining working contexts; employees with motives to craft for their development did so whether or not they worked in supportive contexts. Nevertheless, the informants also expressed a need for support from colleagues and management when engaging in crafting strategies to promote their well-being as well as the common good. When focusing on the patients in their crafting strategies, respondents expressed that work was perceived as more meaningful. Lastly, to increase or maintain manageability in work, job crafters sometimes choose to craft less, or not at all.
